# Association of glucagon-like peptide-1 receptor agonists (GLP-1 RAs) and neurogenesis: a systematic review

**DOI:** 10.1017/neu.2025.4

**Published:** 2025-02-14

**Authors:** Hezekiah C.T. Au, Yang Jing Zheng, Gia Han Le, Sabrina Wong, Kayla M. Teopiz, Angela T.H. Kwan, Hartej Gill, Sebastian Badulescu, Kyle Valentino, Joshua D. Rosenblat, Rodrigo B. Mansur, Roger S. McIntyre

**Affiliations:** 1 Brain and Cognition Discovery Foundation, Toronto, Ontario, Canada; 2 Mood Disorder Psychopharmacology Unit, University Health Network, Toronto, Ontario, Canada; 3 Institute of Medical Science, University of Toronto, Toronto, Ontario, Canada; 4 Department of Pharmacology and Toxicology, University of Toronto, Toronto, Ontario, Canada; 5 Faculty of Medicine, University of Ottawa, Ottawa, Ontario, Canada; 6 Department of Psychiatry, University of Toronto, Toronto, Canada

**Keywords:** Glucagon-Like Peptide, GLP-1 receptor agonist, neurogenesis, obesity, Semaglutide, Liraglutide, Exentaide, Tirzepatide, Lixisenatide

## Abstract

**Objective:**

Glucagon-like peptide-1 (GLP-1) and glucagon-like peptide-1 receptor agonist (GLP-1 RA) administration has been associated with neuroproliferative effects and modulatory effects in neuronal pathways. Herein, we conducted a comprehensive synthesis of the effects of GLP-1 and GLP-1 RAs on neurogenesis.

**Methods:**

We examined studies that investigate changes in neurogenesis mediated by GLP-1 and GLP-1 RA administration in both human and animal populations. Relevant articles were retrieved through OVID (MedLine, Embase, AMED, PsychINFO, JBI EBP Database), PubMed, and Web of Science from database inception to July 2nd. Primary studies investigating the role of GLP-1 and GLP-1 RAs on neurogenesis were included for analysis.

**Results:**

GLP-1 and GLP-1 RAs (i.e. exenatide, geniposide, liraglutide, lixisenatide, and semaglutide), increased neurogenesis within the dentate gyrus, hippocampus, olfactory bulb, and the medial striatum in animal models. Additionally, GLP-1 and GLP-1 RAs were associated with modulating changes in multiple apoptotic pathways and upregulating survival pathways.

**Discussion:**

GLP-1 and GLP-1 RAs are positively associated with neurogenesis. This effect may have translational implications insofar as disparate mental disorders that are characterised by neurogenesis defects (e.g. depressive disorders and neurocognitive disorders) may be benefitted by these agents.

Summations

- Glucagon-like peptide-1 and its receptor agonism was positively associated with neurogenesis.
- Changes in neurogenesis were observed in the hippocampus, dentate gyrus, olfactory bulb, and medial striatum.

Considerations

- No human studies were identified, limiting the ability to extend the findings to humans.
- Animal models vary in species and disease models, which may introduce confounding effects.
- Markers for neurogenesis of disparate neuronal populations varied across studies, which may impact the consistency of the results.

## Introduction

Glucagon-like peptide-1 (GLP-1) is a gut-derived incretin hormone indicated for antidiabetic therapy by promoting insulin secretion and inhibition of glucagon secretion (Lutz & Osto, 2016). GLP-1 receptors are broadly distributed both peripherally (e.g., on pancreatic β cells) and within the central nervous system (Lee & Lee, 2017; Muscogiuri *et al*., 2017). Extant literature has reported that GLP-1 receptors are expressed on neurons in regions such as the paraventricular nucleus, hippocampus, and preproglucagon cells in the olfactory bulb (Katsurada *et al*., 2014; Montaner *et al*., 2024; Canário *et al*., 2024).

Neurogenesis is a process described as the formation of neurons through stem and progenitor cell proliferation, occurring mainly in the subgranular zone within the dentate gyrus of the hippocampus and subventricular zone (SVZ) of lateral ventricles (Cope & Gould, 2019; Chen *et al.,*2023a). This process is characterised by the proliferation and differentiation of neural stem cells via symmetrical division to form new neural stem cells, and asymmetric division to produce radial glial cells (Shimojo *et al*., 2011). The newly proliferated radial glial cells can further divide into neuroblasts and astrocytes through asymmetric division, subsequently integrating into existing neural circuits (Shimojo *et al*., 2011; Braun & Jessberger, 2014; Ihunwo *et al*., 2016). Neuronal differentiation and proliferation involves the cAMP Response Element-Binding Protein (CREB) and Notch signalling pathways (Merz *et al*., 2011; Bagheri-Mohammadi, 2021). These processes are modulated by various kinases, including protein kinase A (PKA) and phosphoinositide 3-kinase (PI3K) (Kang *et al*., 2003; Bagheri-Mohammadi, 2021).

Extant literature identified that GLP-1 and GLP-1 receptor agonists (GLP-1 RAs) can exert neuroproliferative effects whilst being associated with increased neurogenesis in preclinical models (Velmurugan *et al*., 2012; McIntyre *et al*., 2013; Vaccari *et al*., 2021). It is hypothesised that GLP-1 subserves neuroproliferative effects through the action of PKA and PI3K pathways (Li *et al*., 2010). Notably, the binding of GLP-1 RAs result in increases in cAMP and PI3K, wherein the activation of these secondary messengers lead to activation of factors such as CREB and the Notch signalling pathways, resulting in increased synaptic plasticity and neurogenesis (Kang *et al*., 2003; Bagheri-Mohammadi, 2021; Dworkin & Mantamadiotis, 2010; Ren *et al*., 2021). Notwithstanding, the role of GLP-1 and GLP-1 RAs on neurogenesis has not been adequately explored.

Herein, we examine the effects of GLP-1 RAs on neurogenesis across both preclinical and clinical paradigms. Our goal is to provide a comprehensive update on the impact of each GLP-1 RA in neurogenesis, while highlighting their potential therapeutic applications in neurodegenerative diseases such as Alzheimer’s Disease (AD).

## Methods

### Search strategy

The Preferred Reporting Items for Systematic Reviews and Meta-Analyses (PRISMA) was utilised to conduct this study (Page *et al.,*^2021^). Relevant articles were systematically searched using Web of Science, OVID (MedLINE, Embase, AMED, PsychInfo, JBI EBP), and PubMed from database inception to June 26, 2024. The search string used for the search included: (“GLP-1” OR “Glucagon-Like Peptide-1” OR “Glucagon-Like Peptide 1” OR “GLP-1 Agonist” OR “Glucagon-Like Peptide-1 Agonist” OR “Glucagon-Like Peptide 1 Agonist” OR “Semaglutide” OR “Ozempic” OR “Rybelsus” OR “Wegovy” OR “Dulaglutide” OR “Trulicity” OR “Exenatide” OR “Byetta” OR “Bydureon” OR “Liraglutide” OR “Lixisenatide” OR “Tirzepatide”) AND (“Neurogenesis” OR “Neuron* Proliferation). Separate searches were conducted on Google Scholar and from reference lists to ensure all articles relevant to the topic were captured.

### Study selection and inclusion criteria

Articles obtained from the systematic search were screened through the Covidence platform, wherein duplicate articles were removed (Covidence, 2024). Two reviewers (H.A. and Y.J.Z.) independently screened the titles and abstracts based on the inclusion and exclusion criteria (Table 1). Primary articles that reported on changes in neurogenesis as a result of GLP-1 prescription or administration were retrieved for full-text screening by two reviewers (H.A. and Y.J.Z.) (Table 1). All conflicts were resolved via discussion, and articles deemed eligible by both reviewers were selected for data extraction.

**Table 1.** Eligibility criteria

Inclusion criteria	A primary study, Measurement of neurogenesis, Full-text article available online, Animal Studies, English language.
Exclusion criteria	Non-primary or secondary research (i.e., literature reviews, systematic reviews, meta-analyses, posters, abstracts, guidelines, protocols and theses), Case Reports, Reports an association without statistics, Full-Text is not available.

### Data extraction

A piloted data extraction template was used to organise and obtain data from included studies. Information to be extracted was established *a priori*, including (1) author, (2) study type, (3) sample size, (4) stains, (5) outcomes of interest. Two independent reviewers (H.A. and Y.J.Z.) conducted data extraction, wherein all conflicts were resolved through discussion. Outcomes of interest pertained to changes in neurogenesis associated with GLP-1 prescription or administration.

### Quality assessments

Quality assessments were conducted using the SYRCLE’s risk of bias analysis tool for animal studies (Hooijmans *et al*., 2014). Relevant literature was assessed by two independent reviewers (H.A. and Y.J.Z.), wherein the risk of bias was evaluated, and all conflicts were resolved following discussions. Further information on inclusion and exclusion criteria, as well as a summary table, can be found as supplementary material (Tables 1 and 2).

**Table 2.** Characteristics of studies examining effect of GLP-1 and GLP-1 RAs on neurogenesis in animal models

Author(s)	Study type	Sample size	Stains	Outcome(s) of interest
**GLP-1**				
Lennox *et al.*, (2013)	Animal study	Male Swiss TO mice	BrdU	Daily injection of (Val^8^)GLP-1-Glu-PA for 21 days resulted in an 30% increase (*p* < 0.05) in BrdU positive cells within the granular cell layer of the dentate gyrus in comparison to saline controls.
McGovern *et al*. (2012)	Animal study	C57Bl/6J mice 24 Sprague–Dawley rats	**Mature neurons:** BrdU **Immature neurons:** DCX	Mice treated with (Val^8^)GLP-1 for 21 days exhibited a significantly increased number of mature neurons in the dentate gyrus (*p* < 0.05), observed through BrdU staining. Mice treated with (Val^8^)GLP-1 for 21 days exhibited a significantly increased number of immature neurons in the dentate gyrus (*p* < 0.05), observed via DCX staining.
Sampedro *et al*. (2019)	Animal study	45 total mice 30 diabetic *ob/ob* BKS.Cg-Dock7m +/+ Leprdb/J mice 15 non-diabetic BKS.Cg-Dock7m +/+ Leprdb/J mice	Ki67	GLP-1 administration in diabetic mice (*n* = 4) resulted in significant increases in Ki67-positive cells in the ganglion cell layer, inner nuclear layer, inner plexiform layer, outer nuclear layer, and the outer plexiform layer (*p* < 0.05) in comparison to the control group (*n* = 4). GLP-1 administration was associated with downregulation of nuclear factor kappa B (NF-κb) (*p* < 0.05) and VEGF overexpression. In contradistinction, GLP-1 administration was associated with upregulation of survival pathways in glycogen synthase kinase-3 beta (GSK3β) (*p* < 0.05) and B-cell lymphoma-extra-large protein (Bcl-xL) (*p* < 0.05)
**Exenatide**				
Belsham *et al*. (2009)	Animal study	Wild-type C57BL/6 male mice 14 GLP-1 receptor knockout mice	BrdU Ki67	Exendin-4 treatment over a one-week period led to a twofold increase in BrdU-positive cells, with results replicated by Ki67 immunostaining (*p* < 0.05). Administration of exendin 9-39, a GLP-1 receptor antagonist resulted in 80% decrease in Ki67 immunostaining in comparison to vehicle controls (*p* < 0.01).
Bertilsson *et al.* (2007)	Animal study	C57 black mice Male Wistar rats	**Mature neurons:** BrdU **Neuroblasts:** DCX	Rats administered with Ex-4 twice per day over a 7-day period exhibited a near twofold increase (*p* < 0.01) in BrdU-positive cells in the subventricular zone. Rats administered with Ex-4 twice per day over a 7-day period exhibited a 70% increase in DCX-positive cells (*p* < 0.01) in the medial striatum in comparison to vehicle controls.
Darsalia *et al*. (2012)	Animal study	42 male diabetic GK rats	**Mature neurons:** BrdU, NeuN **Immature neurons:** Ki67 **Neuroblasts:** DCX	Analysis using NeuN and BrdU identified no significant changes on neuronal count post-Ex-4 treatment in comparison to PBS in the striatum, subgranular zone, and granular cell layer of the dentate gyrus. Using Ki67 staining, it was observed that Ex-4 treatment over two weeks after stroke resulted in a twofold increase of proliferating cells in the subventricular zone of the hippocampus and dentate gyrus in comparison with PBS saline group (*p* < 0.05). At 4 weeks, no differences were observed between the two groups. Quantification of DCX-positive neuroblasts indicated a 50% increase in neuroblast production after Ex-4 treatment in comparison with the PBS-treated group two weeks after stroke (*p* < 0.05). This effect was not observed after four weeks. Concentration of Ex-4 administered did not have a significant effect on neurogenesis.
Hamilton *et al*. (2011)	Animal study	Male Swiss TO mice Aston mice	**Mature neurons:** BrdU **Developing neurons:** DCX	Ex-4 treated *ob/ob* mice exhibited a 65% increase in BrdU-positive cells in comparison to saline treated controls (*p* < 0.001). Liraglutide treated *ob/ob* mice exhibited a 50% increase in BrdU-positive cells in comparison to saline treated controls (*p* < 0.001). Ex-4 treated *db/db* mice exhibited a 63% increase in BrdU-positive cells in comparison to saline treated controls (*p* < 0.001). Liraglutide treated *db/db* mice exhibited a 88% increase in BrdU-positive cells in comparison to saline treated controls (*p* < 0.001). High-fat diet mice treated with Ex-4 had significantly more DCX-positive neurons in comparison to saline-treated HF mice (*p* < 0.001). High-fat diet mice treated with liraglutide had significantly more DCX-positive neurons in comparison to saline-treated HF mice (*p* < 0.001).
Pathak *et al*. (2018)	Animal study	DIH Swiss mice	DCX	Administration of Ex-4 resulted in significantly increased DCX-positive cells in the hippocampus when compared to controls (*p* < 0.001).
Solmaz *et al*. (2015)	Animal study	21 Sprague-Dawley albino male mature rats	N/A	Mice treated with streptozotocin and exenatide exhibited significantly increased neuronal count in the hippocampus when compared to mice treated with streptozotocin and saline (*p* < 0.05).
**Geniposide**				
Sun *et al*. (2021)	Animal Study	C57BL/6 J mice	DCX	Geniposide, a GLP-1 receptor agonist, significantly increased the number of DCX^+^ cells (*p* < 0.05) and increased the number of DCX^+^ dendrites (*p* < 0.05), suggesting growth of developing neurons.
**Liraglutide**				
Parthsarathy and Hölscher (2013)	Animal study	96 total mice 48 female APP_SWE_/PS1_DE9_ mice 48 wild-type littermate mice	**Mature neurons:** BrdU/NeuN **Immature neurons:** Ki67 DCX	**Acute liraglutide treatment** Acute treatment was characterized by 7 continuous days of liraglutide administration. Wild-type mice treated with liraglutide did not show an increase in newly generated cells in the dentate gyrus in comparison to animals treated with saline at 3, 6, 12, and 15 months of age (*p* > 0.05) when using BrdU as a marker. However, increased cell proliferation was observed after liraglutide treatment in comparison to saline at 3 months (90%), 6 months (63%), 12 months (114%), 15 months (137%) of age (*p* < 0.05) when using Ki67 immunostaining. Similarly, an increase in DCX-positive cells was observed after liraglutide treatment in comparison to saline at 3 months (59%), 6 months (26%), 12 months (57%), and 15 months (61%) of age (*p* < 0.05). AD mice treated with liraglutide exhibited increased cell proliferation in the dentate gyrus in comparison to saline at 3 months (33%; *p* < 0.05), 6 months (41%; *p* < 0.05), 12 months (69%; *p* < 0.01), and 15 months (65%; *p* < 0.05) of age (*p* < 0.05) when using BrdU as a marker. Similarly, increased cell proliferation was observed after liraglutide treatment in comparison to saline at 3 months (99%), 6 months (58%), 12 months (153%), 15 months (135%) of age (*p* < 0.05) when using Ki67 immunostaining. Similarly, an increase in DCX-positive cells after liraglutide treatment in comparison to saline at 3 months (50%), 6 months (33%), 12 months (53%), and 15 months (72%) of age (*p* < 0.05). In acute treatment groups, a non-significant increase in neurogenesis was observed post-liraglutide treatment in comparison to saline controls at 3 months (8%), 6 months (21%), 12 months (20%), and 15 months (22%) of age in wild-type mice (*p* > 0.05). Similarly, a non-significant trend in neurogenesis was observed in AD mice models, wherein an increase was observed in the liraglutide group comparison to saline at 3 months (19%), 6 months (18%), 12 months (21%), and 15 months (12%) of age (*p* > 0.05). No significant changes in gliogenesis were observed in wild-type mice treated with liraglutide (*p* > 0.05). **Chronic liraglutide treatment** Chronic treatment was characterized by 37 continuous days of liraglutide administration. Significant increases in cell proliferation in the dentate gyrus was observed in wild-type mice when administered with liraglutide in comparison to saline controls at 3 months (20%; *p* = 0.0457), 6 months (22%; *p* = 0.0467), 12 months (36%; *p* = 0.0455), and 15 months (52%; *p* < 0.05) of age when using BrdU stain. Similarly, increased proliferation was observed post-liraglutide treatment in comparison to saline at 3 months (94%), 6 months (103%), 12 months (143%), and 15 months (122%) of age when using Ki67 immunostaining (*p* < 0.05). An increase in DCX-marked immature neurons post-liraglutide treatment in comparison to saline controls was also observed at 3 months (43%), 6 months (40%), 12 months (74%), and 15 months (68%) of age (*p* < 0.05). AD mice chronically treated with liraglutide also exhibited increased proliferation in the dentate gyrus with respect to saline controls at 3 months (55%; *p* < 0.01), 6 months (50%; *p* < 0.05), 12 months (68%; *p* < 0.01), and 15 months (79%; *p* < 0.01) of age when using BrdU stain. Similarly, increased proliferation was observed post-liraglutide treatment in comparison to saline at 3 months (175%), 6 months (70%), 12 months (215%), and 15 months (120%) of age when using Ki67 immunostaining (*p* < 0.01). An increase in DCX-marked immature neurons post-liraglutide treatment in comparison to saline controls were also observed at 3 months (35%), 6 months (48%), 12 months (88%), and 15 months (94%) of age (*p* < 0.05). No significant changes in gliogenesis were observed in either group of mice treated with liraglutide (*p* > 0.05).
Salles *et al*. (2018)	Animal study	Female APP/PS1 mice C57BL/6 mice	Astrocytes: GFAP Neuroblasts: DCX	Administration of liraglutide was not significantly associated with changes in astrocyte numbers (*p* > 0.05), but was associated with increases in DCX-marked neuroblasts in subventricular zone of wild-type (*p* < 0.05) and APP/PS1 (*p* < 0.01) in comparison to saline treatment.
Weina *et al*. (2018)	Animal study	Adult male C57BL/6N mice	DCX	Mice were injected with corticosterone to induce depressive symptoms over thirty days. Mice injected with 20 nm/kg liraglutide had significantly increased density of DCX-positive immature neurons in comparison to saline treatment (*p* = 0.0457)
Zhao *et al*. (2022)	Animal study	40 female C57BL/6 mice	Ki67	Ki67 staining identified significant increases in neuron count (*p* < 0.05) in the dentate gyrus after liraglutide administration over 8 weeks in comparison to saline treatment over the same period of time. Liraglutide administration was associated with increased activation of Wnt/β-catenin signaling pathways (*p* < 0.05)-catenin signaling pathways (*p* < 0.05)
**Lixisenatide**				
Hunter and Hölscher (2012)	Animal study	C57BL/6 mice	BrdU DCX	Liraglutide administration was associated with significant increase in cAMP levels (*p* < 0.05). An 80% increase in neuronal proliferation was observed through BrdU analysis (*p* < 0.01) in lixisenatide groups in comparison to saline groups. A 70% increase in young neuronal count was observed via DCX analysis (*p* < 0.05) in lixisenatide groups in comparison to saline groups.
Ren *et al*. (2021)	Animal study	C57BL/6N mice	BrdU DCX	Intranasal lixisenatide treatment at 10mg/kg/d and 50mg/kg/d increased the number of BrdU and BrdU/DCX marked cells in the olfactory bulb and hippocampus (*p* < 0.001).
**Semaglutide**				
Yang *et al*. (2019)	Animal study	144 Male Sprague–Dawley rats	**Immature neurons:** DCX **Microglia:** Iba1	Mice with MCAO administered with semaglutide had a significantly higher number of DCX-positive cells in comparison to mice with MCAO administered with saline (*F* = 25.277, *p* < 0.01). Treatment of semaglutide significantly reduced the number of Iba1-positive cells in the hippocampus in comparison to saline treatment in ischemic animals and mice with MCAO (*p* < 0.001). Expression of neurogenesis markers nestin, CXCR4, and SDF-1 were greater in mice with MCAO administered with semaglutide in comparison to mice with MCAO administered with saline (*p* < 0.001) Administration of semaglutide was associated with significant changes in apoptotic pathways. Increases in proto-oncogene c-RAF (c-Raf) (*p* < 0.05), mitogen-activated protein kinase 1 (ERK2) (*p* < 0.01), B-cell lymphoma-2 (Bcl-2) (*p* < 0.05), and decreases in Caspase-3 (*p* < 0.05) were observed one week after administration.

BrdU, Bromodeoxyuridine; DCX, Doublecortin; Ki67, Antigen Kiel 67; NeuN, Hexaribonucleotide Binding Protein-3; Ex-4, Exendin-4; MCAO, Middle Cerebral Artery Occlusion; Iba1, Ionized Calcium-binding Adapter.

## Results

### Search results

**Figure 1.**
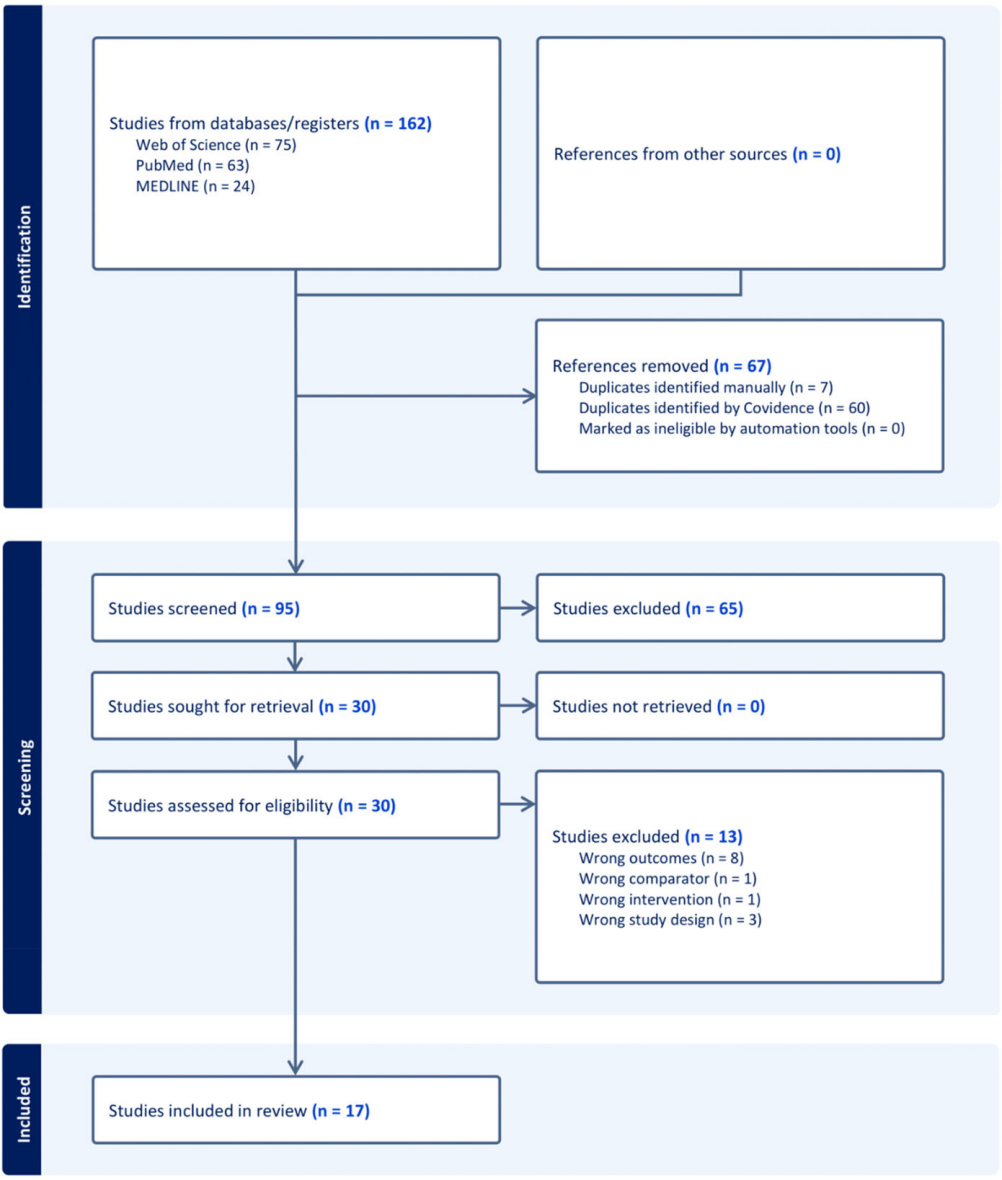
PRISMA flow diagram of literature search ( Covidence, 2024).

A systematic search generated a total of 162 studies, wherein 7 duplicates were identified manually, and 60 duplicates were identified by Covidence. 95 studies underwent abstract and title screening, with 65 articles deemed irrelevant. In accordance with the inclusion and exclusion criteria, 30 full-text studies were assessed for eligibility, of which, 13 were excluded due to wrong outcomes (*n* = 8), wrong study designs (*n* = 3), wrong comparator (*n* = 1), and wrong intervention (*n* = 1), yielding a total of 17 studies for further analysis (Figure 1, Table S2). Although animal and human studies were eligible for inclusion, the search only yielded animal studies.

### Methodological quality

Quality assessment of the included studies was conducted using the SYRCLE’s risk of bias tool for animal studies (Hooijmans *et al*., 2014). Studies utilizing animal cell culture generated ‘not reported’ (NR) or ‘X’ notations, as some prompts were not applicable or not reported within the studies. However, as these studies derive cell culture from live animals, the SYRCLE’s risk of bias tool was used.

All of the included studies assessed preclinical literature, demonstrating low attrition and reporting bias. Common limitations of selected studies include insufficient detection bias and blinding procedures, including allocation concealment, random housing, and random outcome assessment domains.

The bias assessment in animal model studies revealed distinct patterns that correspond to their overall quality ratings. Studies assessed as “Good,” such as those conducted by Belsham *et al*., (2009); McGovern *et al*., (2012); Pathak *et al*., (2018); Sampedro *et al*., (2019); Solmaz *et al*., (2015); Weina *et al*., (2018); and Yang *et al*., (2019), generally exhibit a lower risk of bias with most items adequately reported, despite occasional limitations in areas like random housing or random outcome assessment. In contradistinction, studies rated as ‘Fair’, including those by Bertilsson *et al.,*(2007), Darsalia *et al*., (2012); Hamilton *et al*., (2011), Lennox et al., (2013), Parthsarathy & Hölscher (2013), Ren *et al*., (2021); and Zhao *et al*., (2022), often show a higher number of items marked as “Not Reported” or “No,” particularly in baseline characteristics, allocation concealment, and random housing. Additionally, the study by Hunter & Hölscher (2012) was rated ‘‘Poor’’ as it consistently demonstrate significant bias with multiple items inadequately reported, highlighting the necessity for rigorous methodology to ensure comparability and reliability in animal research (Table S1). This pattern emphasises the link between comprehensive reporting and a lower risk of bias, underscoring the crucial significance of thorough methodological transparency in producing high-quality, reliable scientific outcomes.

### GLP-1 effects on neurogenesis in animal models

We have identified three studies examining the effects of GLP-1 and neurogenesis (Table 2) (Lennox *et al*., 2013; McGovern *et al*., 2012; Sampedro *et al*., 2019). Lennox et al., (2013) identified an association between (Val^8^)GLP-1-Glu-RA administration and a 30% increase in BrdU-positive cells (*p* < 0.05) within the granular cell layer of the dentate gyrus of mice in comparison to saline controls (Lennox *et al*., 2013). The increase in BrdU-positive cells indicate a significant difference in mature neuronal populations between GLP-1 administration and saline controls (Lennox *et al*., 2013). This trend was in accordance with results from McGovern *et al*., (2012), wherein (Val^8^)GLP-1-Glu-PA administration was significantly associated with increases in BrdU-positive cells (*p* < 0.05) and DCX-positive cells (*p* < 0.05) in mice dentate gyrus (McGovern *et al*., 2012). Similarly, Sampedro *et al*., (2019) reported significant increases in neurogenesis ascertained by marker Ki-67 in the ganglion cell layer, inner nuclear layer, inner plexiform layer, outer nuclear layer, and the outer plexiform layer (*p* < 0.05) of obese mice treated with GLP-1 in comparison to controls (Sampedro *et al*., 2019). Notably, GLP-1 administration was also associated with upregulation of survival pathways glycogen synthase kinase-3 beta (GSK3β) (*p* < 0.05) and B-cell lymphoma-extra-large protein (Bcl-xL) (*p* < 0.05) (Sampedro *et al*., 2019). Taken together, these results suggest that GLP-1 increases neurogenesis in various regions of the brain and provides mechanistic insight on the pathways in which GLP-1 can modulate neurogenesis (Lennox *et al*., 2013; McGovern *et al*., 2012; Sampedro *et al*., 2019).

### Association between GLP-1 RAs and neurogenesis in animal models

To understand the effects of GLP-1 RAs and neurogenesis in animal models, we have identified 13 studies (Table 2). Of the studies included herein, Hamilton *et al*., (2011) characterised the effect of both exenatide and liraglutide on neurogenesis.

### Effect of exenatide on neurogenesis in animal models

Findings from Solmaz *et al*., (2015) identified significantly higher hippocampal neuronal count (*p* < 0.05) in mice treated with exenatide in comparison to saline (Solmaz *et al*., 2015). These results were replicated by Pathak *et al*., (2018), wherein exendin-4 (Ex-4) administration in mice was associated with significantly increased DCX-positive cell count in comparison to controls (*p* < 0.001). The aforementioned trends accord with the results by Darsalia *et al*., (2012) wherein Ex-4 treatment over two weeks was associated with a 50% in neuroblast production when compared to PBS-treated rats (*p* < 50%).

Furthermore, analysis using Ki67 staining identified that Ex-4 treatment over two weeks was associated with a two-fold increase in proliferating cells in the SVZ of the hippocampus and dentate gyrus (*p* < 0.05) (Darsalia *et al*., 2012). However, no effects of Ex-4 were reported when a NeuN/BrdU double marker was used to examine the effects of Ex-4 treatment (Darsalia *et al*., 2012). Notwithstanding, Hamilton *et al*., (2011) reported a 65 and 63% increase in BrdU-positive cells in Ex-4 treated obese (*ob/ob)* mice and diabetic (*db/db)* mice (mice with a point mutation causing leptin receptor deficiency) (*p* < 0.001) in comparison to saline controls, respectively (Hamilton *et al*., 2011; Suriano *et al*., 2021). High-fat diet mice treated with Ex-4 also exhibited a significant increase in DCX-positive neurons in comparison to saline-treated mice (*p* < 0.001) (Hamilton *et al*., 2011). These trends are further reinforced by findings from Bertilsson *et al.,* (2007), wherein *bis in die* administration of Ex-4 for 7 days in rats was associated with a near two-fold and 70% increase in BrdU-positive cells in the SVZ and DCX-positive cells in the medial striatum, respectively (*p* < 0.01) (Bertilsson *et al*., 2007). Consistent with the aforementioned trends, results from Belsham *et al*., (2009) identified that Ex-4 treatment over a one-week period was associated with a two-fold increase in BrdU-positive cells (*p* < 0.05) in mice (Belsham *et al*., 2009). These findings suggest the presence of a positive association between exenatide and neurogenesis.

### Geniposide effect on neurogenesis in animal models

There is currently insufficient information on the effects of geniposide on neurogenesis. Notwithstanding, an experimental study by Sun *et al*., (2021) examined the effect of geniposide in mice. Findings suggest that geniposide mediated an increase in DCX-positive cells and dendrites in mice treated with geniposide in comparison to controls (*p* < 0.05). Due to the limited data available, a comprehensive evaluation of geniposide’s effect on neurogenesis cannot be made.

### Liraglutide effect on neurogenesis in animal models

We identified 6 studies examining the role of liraglutide in neurogenesis (Hamilton *et al*., 2011; Hunter & Hölscher, 2012; Parthsarathy & Hölscher, 2013; Weina *et al*., 2018; Salles *et al*., 2018; Zhao *et al*., 2022). Weina *et al*., (2018) reported a significant increase in the density of DCX-positive immature neurons in mice injected with corticosterone and liraglutide in comparison with mice injected with corticosterone and saline (*p* = 0.0456). Additionally, *ob/ob* mice treated with liraglutide exhibited a 65% increase in BrdU-positive cells, wherein *db/db* mice treated with liraglutide exhibited an 88% increase in BrdU-positive cells in comparison to saline controls (*p* < 0.001) (Hamilton *et al*., 2011). Similarly, a significant increase in neuronal count in the dentate gyrus of mice injected with liraglutide in comparison to controls (*p* < 0.05) was observed (Zhao *et al*., 2022). Simultaneously, liraglutide administration was also associated with increased activation of the Wnt/β-catenin pathway (*p* < 0.05) (Zhao *et al*., 2022). Additionally, liraglutide administration was associated with significant increases in cAMP levels in comparison to controls (*p* < 0.05) (Hunter & Hölscher, 2012). In a separate study by Salles *et al*., (2018), liraglutide administration was associated with increases in DCX-marked neuroblasts of wild-type mice (*p* < 0.05) and APP/PS1 mice (*p* < 0.01) in the SVZ when compared to saline treatment. In contradistinction, liraglutide did not significantly change levels of GFAP-marked astrocytes (*p* > 0.05) (Salles *et al*., 2018).-catenin pathway (*p* < 0.05) (Zhao *et al*., 2022). Additionally, liraglutide administration was associated with significant increases in cAMP levels in comparison to controls (*p* < 0.05) (Hunter & Hölscher, 2012). In a separate study by Salles *et al*., (2018), liraglutide administration was associated with increases in DCX-marked neuroblasts of wild-type mice (*p* < 0.05) and APP/PS1 mice (p < 0.01) in the SVZ when compared to saline treatment. In contradistinction, liraglutide did not significantly change levels of GFAP-marked astrocytes (*p* > 0.05) (Salles *et al*., 2018).

Parthsarathy & Hölscher (2013) identified that wild-type mice treated with liraglutide over a week exhibited increased cell proliferation at 3 months (90%), 6 months (63%), 12 months (114%), and 15 months (137%) of age (*p* < 0.05) in comparison to saline controls. Similarly, an increase in DCX-marked immature neurons was observed after liraglutide treatment in comparison to saline at 3 months (59%), 6 months (26%), 12 months (57%), and 15 months (61%) of age (*p* < 0.05) (Parthsarathy and Hölscher, 2013).

This trend was replicated in wild-type mice treated with liraglutide over 37 days, wherein significant increases in cell proliferation in the dentate gyrus were observed in comparison to saline controls at 3 months (20%; *p* = 0.0457), 6 months (22%; *p* = 0.0467), 12 months (36%; *p* = 0.0455), and 15 months (52%; *p* < 0.05) of age (Parthsarathy & Hölscher, 2013). Similarly, increased proliferation was observed using Ki67 immunostaining post-liraglutide treatment in comparison to saline treatment at 3 months (94%), 6 months (103%), 12 months (143%), and 15 months (122%) of age (Parthsarathy & Hölscher, 2013). Additionally, an increase in DCX-positive neurons post-liraglutide treatment in comparison to saline controls was also observed at 3 months (43%), 6 months (40%), 12 months (74%), and 15 months (68%) of age (*p* < 0.05) (Parthsarathy & Hölscher, 2013). Notwithstanding, no significant change in gliogenesis was observed in mice treated with liraglutide (*p* > 0.05) (Parthsarathy & Hölscher, 2013). Results suggest a positive association between liraglutide and neurogenesis, which may be mediated through changes in the Wnt/β-catenin pathway.-catenin pathway.

### Lixisenatide effect on neurogenesis in animal models

To examine the association between lixisenatide and neurogenesis, we identified two studies (Hunter & Hölscher, 2012; Ren *et al*., 2021). In an experimental study by Hunter & Hölscher (2012), mice treated with lixisenatide exhibited an 80% increase in neuronal proliferation marked by BrdU-positive cells in comparison to control (*p* < 0.01). Additionally, a 70% increase in proliferating neurons was observed in lixisenatide-treated mice in comparison to saline controls observed via DCX analysis (*p* < 0.05) (Hunter & Hölscher, 2012). Similarly, Ren *et al*., (2021) reported that intranasal lixisenatide administration was associated with increased numbers of BrdU and BrdU/DCX-marked cells in the olfactory bulb and hippocampus (*p* < 0.001). Taken together, these results suggest a positive association between lixisenatide and neurogenesis (Hunter & Hölscher, 2012; Ren *et al*., 2021).

### Semaglutide effect on neurogenesis in animal models

There is insufficient evidence on the effect of semaglutide in neurogenesis. Notwithstanding, a study by Yang *et al*., (2019) reported that mice with middle cerebral artery occlusion administered with semaglutide had a significantly higher number of DCX-positive cells in comparison to saline administration (*F* = 25.277, *p* < 0.01) whilst also exhibiting increased expression of neurogenesis markers nestin, CXCR4, and SDF-1 (*p* < 0.01) (Yang *et al*., 2019). Contrastingly, semaglutide administration was also associated with a significant reduction of Iba1-positive cells in the hippocampus in comparison to saline treatment (*p* < 0.001) (Yang *et al*., 2019). Additionally, administration of semaglutide was associated with significant changes in apoptotic pathways (Yang *et al*., 2019). Notably, increases in proto-oncogene c-RAF (c-Raf) (*p* < 0.05), mitogen-activated protein kinase 1 (ERK2) (*p* < 0.01), B-cell lymphoma-2 (Bcl-2) (*p* < 0.05), and decreases in Caspase-3 (*p* < 0.05) were observed one week after semaglutide administration (Yang *et al*., 2019). Although these findings suggest that semaglutide is likely associated with increased levels of neurogenesis, further research is required to investigate changes in specific neuron types.

## Discussion

To our knowledge, this systematic review represents the first comprehensive examination of the association between GLP-1s, GLP-1 RAs, and their effects on neurogenesis. Existing literature consistently reports a positive relationship between GLP-1, specific GLP-1 RAs, and neurogenesis, highlighting their potential significance in this domain.

Overall, our results indicate that GLP-1 administration is associated with increased levels of neurogenesis in the dentate gyrus, ganglion cell layer, inner nuclear layer, inner plexiform layer, outer nuclear layer, and the outer plexiform layer. Furthermore, administration of the GLP-1 RAs exenatide, geniposide, liraglutide, lixisenatide, and semaglutide were associated with increased neurogenesis, mainly in the hippocampus and the dentate gyrus. Additionally, changes in neurogenesis exist alongside antiapoptotic and neuroprotective effects (Darsalia *et al*., 2012; Sampedro *et al*., 2019; Yang *et al*., 2019). Notwithstanding, increased levels of neurogenesis were also observed in the medial striatum and olfactory bulb, suggesting that GLP-1 RAs may be associated with modulating neurogenesis outside of the hippocampus and dentate gyrus.

Additionally, our results identified that GLP-1 and GLP-1 RA administration are associated with changes in molecular pathways relevant to changes to neurogenesis. Notably, GLP-1 administration was associated with upregulation of GSK3β and Bcl-xL pathways. Similarly, liraglutide was associated with upregulation of the Wnt/β-catenin pathway, whilst increasing cAMP concentrations in the brain (Hunter & Hölscher, 2012; Zhao *et al*., 2022). In contradistinction, semaglutide was associated with elevated activation of Bcl-2, c-Raf, and MEK1 pathways, whilst decreasing activity of Caspase-3 (Yang *et al*., 2019).-catenin pathway, whilst increasing cAMP concentrations in the brain (Hunter & Hölscher, 2012; Zhao *et al*., 2022). In contradistinction, semaglutide was associated with elevated activation of Bcl-2, c-Raf, and MEK1 pathways, whilst decreasing activity of Caspase-3 (Yang *et al*., 2019).

The effects of GLP-1 RA on neurogenesis may be modulated through changes in signaling pathways. Notably, increased activity GSK3β, Bcl-xL, Bcl-2, Wnt/β-catenin, c-Raf, and MEK1 have been associated with the promotion of neuronal differentiation and proliferation, wherein decreased activity of Caspase-3 has been linked to anti-apoptotic effects (Pimentel *et al*., 2000; Hur & Zhou, 2010; Lei *et al*., 2012; Fogarty *et al*., 2016; Aniol *et al*., 2016; Zhang & Liu, 2002; Rogers *et al*., 2017). Additionally, GLP-1 RAs have been noted to exert an anti-inflammatory effect, reducing levels of inflammatory cytokines IL-1β and TNF-α (Zhang *et al*., 2021). This has been noted to exert a protective effect on dopaminergic neurons (Zhang *et al*., 2021). As such, it could be hypothesised that GLP-1 and GLP-1 RAs subserves neurogenesis through multiple molecular and cellular systems (Zhao *et al*., 2022; Hunter & Hölscher, 2012; Yang *et al*., 2019). Alterations within neuronal development and apoptosis are cellular effects that are observed in mood disorders and neurodegenerative diseases (Martins-Macedo *et al*., 2021; Chen *et al.,* 2023b). As such, GLP-1 RAs may serve as an effective option in treating neurodegenerative diseases for persons with obesity. By understanding the association between GLP-1 and GLP-1 RAs on neurogenesis, we are able to examine how these agents can facilitate the regeneration of neurons in neurodegenerative diseases such as Alzheimer’s Disease (AD) and Parkinson’s Disease (PD) (Holst *et al*., 2011).-catenin, c-Raf, and MEK1 have been associated with the promotion of neuronal differentiation and proliferation, wherein decreased activity of Caspase-3 has been linked to anti-apoptotic effects (Pimentel *et al*., 2000; Hur & Zhou, 2010; Lei *et al*., 2012; Fogarty *et al*., 2016; Aniol *et al*., 2016; Zhang & Liu, 2002;, Rogers *et al*., 2017). Additionally, GLP-1 RAs have been noted to exert an anti-inflammatory effect, reducing levels of inflammatory cytokines IL-1β and TNF-α (Zhang *et al*., 2021). This has been noted to exert a protective effect on dopaminergic neurons (Zhang *et al*., 2021). As such, it could be hypothesised that GLP-1 and GLP-1 RAs subserves neurogenesis through multiple molecular and cellular systems (Zhao *et al*., 2022; Hunter and Hölscher, 2013; Yang *et al*., 2019). Alterations within neuronal development and apoptosis are cellular effects that are observed in mood disorders and neurodegenerative diseases (Martins-Macedo *et al*., 2021; Chen *et al*., 2023). As such, GLP-1 RAs may serve as an effective option in treating neurodegenerative diseases for persons with obesity. By understanding the association between GLP-1 and GLP-1 RAs on neurogenesis, we are able to examine how these agents can facilitate the regeneration of neurons in neurodegenerative diseases such as Alzheimer’s Disease (AD) and Parkinson’s Disease (PD) (Holst *et al*., 2011). Although it could be hypothesised that changes in neurogenesis mediated by GLP-1 and GLP-1 RA may provide neuroprotective benefits in AD and PD-related pathogenesis, this hypothesis requires testing (Harkavyi *et al*., 2008; Cao *et al*., 2018; Tai *et al*., 2018). Notwithstanding, the aforementioned neuroprotective and neuroproliferative effects modulated by GLP-1 RAs highlights the potential of repurposing these agents as an effective option for weight management in individuals with neurodegenerative conditions.

Interpretations and inferences of our systematic review may be affected by methodological limitations. First, our review identified no human studies investigating the association between GLP-1 and GLP-1 RAs on neurogenesis, limiting our ability to extend our findings to humans. Furthermore, assessed animal models vary in species and disease models, which may confound the interpretation of our results. Moreover, GLP-1 RAs vary in structure and bioavailability, which limits our understanding of the extent to which GLP-1 RAs can exert neurogenesis. Notwithstanding, further research vistas should be directed to identifying the association between GLP-1 and GLP-1 RAs on neurogenesis in different neuronal populations, whilst examining the effect of these agents in neurodegenerative diseases such as AD and PD.

## Conclusion

Herein, we report a positive association between GLP-1, exenatide, geniposide, liraglutide, lixisenatide, and semaglutide on neurogenesis. These findings provide a summary of the association between GLP-1 and GLP-1 RAs on neurogenesis, and provide a foundation for developing GLP-1 and GLP-1 RAs as potential therapeutic strategies for neurodegenerative diseases.

## Supporting information

Au et al. supplementary material Au et al. supplementary material

## Supplementary material

The supplementary material for this article can be found at https://doi.org/10.1017/neu.2025.4.
