# Supplementary material for: Association of glucagon-like peptide-1 receptor agonists (GLP-1 RAs) and neurogenesis: a systematic review
Source: Acta Neuropsychiatr. 2025 Feb 14;37:e50. doi: 10.1017/neu.2025.4 (PMC13130299; doi:10.1017/neu.2025.4)
Supplement: Au et al. supplementary material [file S0924270825000043sup001.docx]

**SUPPLEMENTARY MATERIALS**

**Table S1.** Risk of bias/quality assessment of the included studies using the SYRCLE’s risk of bias tool for animal studies (Hooijmans et al., 2014).

| **Study** | **Item** | | | | | | | | | | **Quality Rating** |
| --- | --- | --- | --- | --- | --- | --- | --- | --- | --- | --- | --- |
|  | **1** | **2** | **3** | **4** | **5** | **6** | **7** | **8** | **9** | **10** |  |
| Belsham et al. (2009) | **X** | **✓** | **✓** | **X** | **✓** | **✓** | **✓** | **✓** | **✓** | **✓** | Good |
| Bertilsson et al. (2008) | **✓** | **✓** | **X** | **NR** | **NR** | **NR** | **X** | **✓** | **✓** | **✓** | Fair |
| Bo et al. (2021) | **X** | **✓** | **X** | **X** | **X** | **X** | **X** | **✓** | **✓** | **✓** | Poor |
| Darsalia et al. (2012) | **NR** | **✓** | **NR** | **NR** | **✓** | **X** | **✓** | **✓** | **✓** | **✓** | Fair |
| Hamilton et al. (2011) | **NR** | **✓** | **X** | **NR** | **X** | **X** | **X** | **✓** | **✓** | **✓** | Fair |
| Hunter and Hölscher (2012) | **✓** | **✓** | **X** | **NR** | **X** | **X** | **X** | **X** | **✓** | **✓** | Poor |
| Lennox et al. (2013) | **✓** | **✓** | **X** | **X** | **X** | **X** | **X** | **✓** | **✓** | **✓** | Fair |
| McGovern et al. (2012) | **X** | **✓** | **X** | **✓** | **NR** | **✓** | **NR** | **✓** | **✓** | **✓** | Good |
| Pathak et al. (2018) | **✓** | **✓** | **✓** | **NR** | **NR** | **NR** | **✓** | **✓** | **✓** | **✓** | Good |
| Parthsarathy and Hölscher (2013) | **X** | **✓** | **X** | **✓** | **NR** | **NR** | **NR** | **✓** | **✓** | **✓** | Fair |
| Ren et al. (2021) | **✓** | **✓** | **X** | **X** | **NR** | **NR** | **NR** | **✓** | **✓** | **✓** | Fair |
| Salles et al. (2018) | **✓** | **✓** | **✓** | **✓** | **NR** | **✓** | **NR** | **✓** | **✓** | **✓** | Good |
| Sampedro et al. (2019) | **✓** | **✓** | **X** | **X** | **✓** | **X** | **✓** | **✓** | **✓** | **✓** | Good |
| Solmaz et al. (2015) | **✓** | **✓** | **✓** | **X** | **✓** | **✓** | **✓** | **✓** | **✓** | **✓** | Good |
| Weina et al. (2018) | **NR** | **✓** | **NR** | **NR** | **✓** | **NR** | **✓** | **✓** | **✓** | **✓** | Good |
| Yang et al. (2019) | **✓** | **✓** | **✓** | **✓** | **✓** | **NR** | **✓** | **✓** | **✓** | **✓** | Good |
| Zhao et al. (2022) | **✓** | **✓** | **X** | **X** | **✓** | **X** | **X** | **✓** | **✓** | **✓** | Fair |

Symbols: ✓ - yes; X - no

Abbreviations: NR = not reported; NA = not applicable

**Table S2.** Studies excluded from data extraction.

| Author(s) | Study DOI | Reason of Exclusion |
| --- | --- | --- |
| **Excluded** | | |
| Abdelkawy et al. (2024) | https://dx.doi.org/10.1016/j.ejphar.2024.176525 | Wrong outcomes.   - No specific neuronal marker was used. Rather, molecular indicators of factors that may be implicated in neurogenesis were used. |
| Augestead et al. (2022) | https://dx.doi.org/10.1111/bph.15524 | Wrong outcomes.   - No specific examination of GLP-1 RAs on neurogenesis. |
| Aviles-Olmos et al. (2012) | https://doi.org/10.1002/mds.25051 | Wrong study design.   - The article is a poster presentation. |
| Coplan et al. (2014) | https://doi.org/10.1155/2014/917981 | Wrong comparator.   - The study does not capture the association of GLP-1 administration on neurogenesis. |
| Diz-Chaves et al. (2022) | https://dx.doi.org/10.1016/bs.vh.2021.12.005 | Wrong study design.   - The article is a book chapter that uses secondary results. |
| Harkavyi et al. (2008) | https://doi.org/10.1186/1742-2094-5-19 | Wrong outcomes.   - The article examines changes in neurotransmitter levels rather than examining changes in cell population, hence out of the scope of this study. |
| Harkavyi et al. (2013) | https://doi.org/10.1155/2013/407152 | Wrong outcomes.   - Outcomes did not examine changes in comparison to a baseline. |
| Imrich et al. (2020) | https://dx.doi.org/10.1007/s13311-020-00896-5 | Wrong study design.   - The study is an abstract. |
| Li et al. (2016) | https://dx.doi.org/10.4103/1673-5374.177742 | Wrong outcomes.   - The study does not have information regarding the association between GLP-1 and neurogenesis. |
| Mansouri et al. (2014) | https://dx.doi.org/10.4172/2155-6156.1000409 | Wrong outcomes.   - The study examines the neuroprotective effects of GLP-1, but not the association between GLP-1 and neurogenesis. |
| Porter et al. (2013) | https://dx.doi.org/10.1038/ijo.2012.91 | Wrong outcomes.   - The study does not examine the role of GLP-1 on neurogenesis. |
| Tai et al. (2018) | https://dx.doi.org/10.1016/j.brainres.2017.10.012 | Wrong intervention.   - The study uses a triple receptor agonist, acting on various other receptors which may introduce confounds to the study. - The treatment used is also outside the scope of the study. |
| Velmurugan et al. (2012) | https://dx.doi.org/10.1111/jnc.12036 | Wrong outcomes.   - The study does not examine the role of GLP-1 on neurogenesis. |
